# Metagenomic Insights into Antimicrobial Resistance in Small-Scale Poultry and Cattle Farms

**DOI:** 10.3390/microorganisms14020438

**Published:** 2026-02-12

**Authors:** Elijah Ayilaran, Agnes Kilonzo-Nthenge

**Affiliations:** Department of Food and Animal Sciences, Tennessee State University, 3500 John A. Merritt Boulevard, Nashville, TN 37209, USA; eayilara@my.tnstate.edu

**Keywords:** antimicrobial resistance (AMR), antimicrobial resistance genes (ARGs), virulence factors, shotgun metagenomics, food animal production, one health

## Abstract

Antimicrobial resistance (AMR) poses a critical challenge to global health, with food animal production systems recognized as significant reservoirs of antimicrobial-resistant bacteria. This study evaluated the prevalence and distribution of antimicrobial resistance genes (ARGs) and virulence factors (VFs) across small-scale poultry and cattle farms. A total of 468 samples (soil, feces, water, and natural land soil) were collected from four farms and analyzed using shotgun metagenomics. *Proteobacteria* (34.91%) were the dominant phylum across environments, followed by *Cyanobacteria* (15.67%), *Actinobacteria* (14.95%), *Firmicutes* (10.57%), and *Bacteroidetes* (8.69%). Tetracycline (33.41%) and beta-lactam (30.30%) resistance genes were the most abundant, with macrolide (9.32%) and aminoglycoside (8.39%) resistance also detected. Both tetracycline and beta-lactam resistance genes were significantly enriched across sample types (*p* < 0.05). The detection of diverse VFs alongside ARGs highlights the pathogenic potential of bacterial communities in these production systems. Collectively, the findings reveal that small-scale animal farms are reservoirs of AMR with implications for public health through foodborne transmission. Targeted surveillance and control measures are necessary to prevent the dissemination of ARGs into the broader food chain and to safeguard both human and animal health.

## 1. Introduction

Antimicrobial resistance (AMR) is a major challenge with severe implications for public health. It has been widely believed that AMR of environmental origin plays a crucial role in the dissemination of AMR in human pathogens [1,2]. The global burden of AMR is projected to cause up to 10 million deaths annually by 2050 if left unchecked [3]. Beyond direct health impacts, AMR also threatens animal production, food safety, and environmental sustainability, making it a multifaceted challenge requiring integrated solutions. The One Health framework, emphasizing the interconnectedness of human, animal, and environmental health, has gained prominence as a guiding principle for understanding and mitigating AMR [4,5,6]. Livestock production environments serve as critical interfaces where antibiotic use in animals meets resistant bacteria that can persist in feces, soil, and water, thereby creating opportunities for transmission to humans through food products, environmental exposure, and water systems.

Agricultural environments play a critical role in shaping the dynamics of antimicrobial resistance (AMR), serving as both reservoirs and transmission pathways for antibiotic-resistant bacteria (ARB) and antibiotic resistance genes (ARGs) [7]. Key components of the agroecosystem, such as soil, manure, and water, are subject to continual inputs including animal waste, fertilizers, and irrigation water, which introduce resistance elements into the environment [8]. Concurrently, outputs such as runoff and manure application amplify the dissemination of ARB and ARGs into surrounding ecosystems [8]. Studies have consistently shown the persistence of resistance genes in animal manure [9], their accumulation in agricultural soils [10,11], and their contamination of surface and groundwater system [9,12]. This environmental resistome is further facilitated by horizontal gene transfer mechanisms, including plasmids, integrons, and transposons, that allows for the movement of resistance traits between commensal and pathogenic bacteria [13]. As a result, resistance genes originating in agricultural settings can be transferred to clinically relevant pathogens, heightening risks for both veterinary and human medicine [7]. Particularly concerning are mobile genetic elements carrying genes that confer resistance to last-resort antibiotics, such as colistin (mcr-1), which have been detected in environmental matrices near animal production sites [14], highlighting the public health implications of AMR evolution within agroecosystems.

Antibiotics are widely used in animal agriculture, primarily for disease treatment, prevention, and growth promotion. Although regulatory measures in the United States have curtailed the use of medically important antibiotics as growth promoters, substantial quantities continue to be administered for prophylactic and therapeutic purposes [15]. Commonly used classes in livestock production include tetracyclines, macrolides, aminoglycosides, and beta-lactams [16,17]. A substantial fraction of these antibiotics is excreted unmetabolized in animal waste, with estimates suggesting that over 75% of some compounds are released into the environment via urine and feces [18]. These residues accumulate in soil, manure, and water, creating selective pressures that promote the enrichment and persistence of resistant microbial populations [19,20]. Importantly, the ecological and public health impacts of such practices are not confined to the farm; antibiotic-resistant bacteria and resistance genes can disseminate through direct animal–human contact, the application of contaminated manure to crops, runoff into water systems, and the broader food production chain, including contamination of animal-derived food products during on-farm production, slaughter and processing, distribution, and retail environments such as grocery stores. These pathways facilitate the dissemination of antibiotic-resistant bacteria and resistance genes to consumers [21]. These interconnected transmission routes heighten the need for a systems-based approach to understanding antimicrobial resistance within agricultural ecosystems.

While much AMR research has focused on large-scale industrial farms due to their high animal densities and intensive antibiotic use, small- and medium-scale farms are emerging as significant yet understudied contributors to the resistance landscape. In the United States, over 90% of livestock farms are classified as small- or medium-scale operations, emphasizing their substantial contribution to national food production and their potential role in antimicrobial resistance dissemination [22]. These farms play a vital role in local food systems and maintain closer contact with communities, potentially increasing human exposure to resistance determinants. Studies have shown that small-scale operations can harbor diverse and clinically relevant ARGs and virulent genes. For example, [23] investigated fecal, oral, environmental (soil and water), and feed samples from small-scale livestock farms and identified virulence genes including *invA*, *iroB*, *spiC*, *pipD* and int1 in *Salmonella* spp. Other studies also confirmed that ARG levels were significantly elevated in manure-amended soils, particularly from poultry farms [13,24]. Despite these findings, data on U.S. small- and medium-scale farms remain scarce, posing concerns as consumer demand for locally raised animal products grows and potential pathways for ARG transmission into the human food chain expand.

Characterizing the microbial community structure alongside ARGs and virulence genes provides deeper insights into AMR dynamics. Dominant bacterial phyla such as *Proteobacteria*, *Actinobacteria*, *Firmicutes*, and *Bacteroidetes* play essential ecological roles in nutrient cycling but are also frequent carriers of resistance determinants [25,26]. Changes in the relative abundance of these phyla across farm components (soil, feces, water) can reflect selective pressures exerted by antibiotics and other farm management practices. Moreover, studying the co-occurrence of ARGs and virulence genes within specific microbial taxa helps to identify high-risk reservoirs and potential pathways of dissemination [27].

Despite various studies evaluating AMR in different environmental matrices in the agroecosystem, limited knowledge is available in small- and medium-scale poultry and cattle farms in the context of the U.S. Therefore, this study aimed to evaluate the potential of small- and medium-scale poultry and cattle farms as reservoirs of ARGs and VGs. Additionally, dominant bacterial phyla were profiled across soil, feces, and water samples from poultry and cattle farms to evaluate microbial community structure.

## 2. Materials and Methods

### 2.1. Sample Collection

Sampling was conducted on four farms located in Tennessee, comprising one poultry farm and three cattle farms. All farms were classified as small- to medium-scale operations based on USDA criteria. The poultry farm housed fewer than 5000 birds, while cattle farms maintained fewer than 200 head of cattle and operated on land areas below 200 acres. These farms represent non-industrial livestock production systems commonly as-sociated with local and regional food supply chains. Soil samples were randomly collect-ed from multiple locations within each farm, including areas proximal to animal housing and manure deposition zones, at a depth of approximately 5 cm. Farms were recruited based on accessibility and willingness of producers to participate, reflecting practical constraints commonly encountered in on-farm environmental studies. Three poultry or cattle fresh feces samples were randomly collected from each farm. Approximately, 50 g of soil and feces sub-samples were collected using sterile spoons from each poultry/cattle farm and put into the sterile sampling bag (Fisher brand, Pittsburgh, PA, USA). For water samples, three bottles of 500 mL drinking water were aseptically collected from the farm’s water containers. A total of 468 samples including soil (*n* = 144), feces (*n* = 144), and water (*n* = 144) from four animal farms, and natural land soil (*n* = 36), were collected for microbial analysis. Natural land soil samples were collected from non-agricultural sites located at least 1 km away from any livestock operation and with no known history of manure application, serving as background reference controls. All samples were labeled with farm identification and date and immediately transported to Food Microbiology and Safety Laboratory with ice and stored at −20 °C until further processing.

### 2.2. DNA Extraction from Soil, Feces, and Water from Animal Farms

Before DNA extraction, each (50 g) of three sub-samples of feces or soil collected from the same farm were initially mixed well, then 10 g of each sub-sample weighed, combined with three sub-samples (30 g) of feces or soil as one sample for DNA extraction. Accordingly, downstream statistical analyses were performed on pooled farm-level samples, and farm was not treated as a repeated or nested factor. The genomic DNA from animal feces and soil was extracted using QIAamp^®^ DNA Stool Mini Kit (Qiagen, Hilden, Germany) and PowerSoil^®^ DNA Isolation Kit (MO BIO Laboratories, Inc., Carlsbad, CA, USA), respectively. Three water subsamples (500 mL/each) from the same farm were also mixed and then concentrated by filtering through a sterile disposable vacuum filter unit with 0.22 µm polyethersulfone (PES) membranes (Fisher Scientific, Pittsburgh, PA, USA). PowerWater^®^ DNA Isolation Kit (MO BIO Laboratories, Inc., Carlsbad, CA, USA) was used to extract DNA from concentrated water samples. DNA concentration was determined by a NanoDropTM 2000 Spectrophotometer (Thermo Fisher Scientific, Wilmington, DE, USA) and DNA integrity was confirmed using agarose gel electrophoresis as described [28].

### 2.3. Metagenomic Sequencing and Bioinformatics Analysis

DNA (100 ng/sample) of 55 samples (feces = 22; soil= 18; water =13 from poultry and cattle farms and 2 samples from natural land soil) were used to construct fragment libraries. Illumina TruSeq libraries were prepared from genomic DNA and sequenced on Illumina HiSeq system by CosmosID metagenomic software (CosmosID Inc., Rockville, MD, USA) to reveal associated microbial community composition as described elsewhere [29,30,31]. Briefly, the system utilizes a high-performance data-mining k-mer algorithm and highly curated dynamic comparator databases (Kraken GenBook^®^) that rapidly disambiguate millions of short reads into the discrete genomes or genes engendering the particular sequences. For each sample, the reads from a species were assigned to the strain with the highest aggregation statistics. An abundance score for each identified taxa was calculated based on the number of organism-specific k-mers, and their average observed frequency and then normalized to the average percentage of the organism-specific k-mer hit in the samples.

### 2.4. Statistical Analysis

The data were captured in Microsoft Excel spreadsheet (Microsoft Corporation, Redmond, WA, USA) for analysis. Overall comparisons were conducted with sample type (soil, feces, and water) as the main factor. Farm type (poultry vs. cattle) was evaluated through stratified comparisons rather than included as a separate model factor. The relative abundance of phyla, ARGs, and VGs were expressed as percentages. The relative abundance data were analyzed by analysis of variance and chi-square test using SAS (Software (version 9.4; SAS Institute Inc., Cary, NC, USA, 2013).). Normality and homogeneity of variance were assessed prior to ANOVA, and expected cell counts met requirements for chi-square analysis. *p* values of less than 0.05 were considered statistically significant (*p* < 0.05).

Metagenomic sequencing reads were directly analyzed by CosmosID bioinformatics software package. Because shotgun metagenomic data were analyzed using the CosmosID k-mer-based pipeline, which provides normalized relative abundance estimates without OTU clustering, amplicon-specific normalization and differential abundance tools such as rarefaction, DESeq2, or ANCOM were not applied. Multiple one-way ANOVA was used to compare mean relative abundances of bacterial phyla, ARGs, and virulence genes across sample types, whereas chi-square tests were applied to assess differences in categorical resistance and virulence gene distributions.

## 3. Results

### 3.1. Phylogenetic Profile of the Bacterial Community in Poultry and Cattle Farms

**Figure 1:**
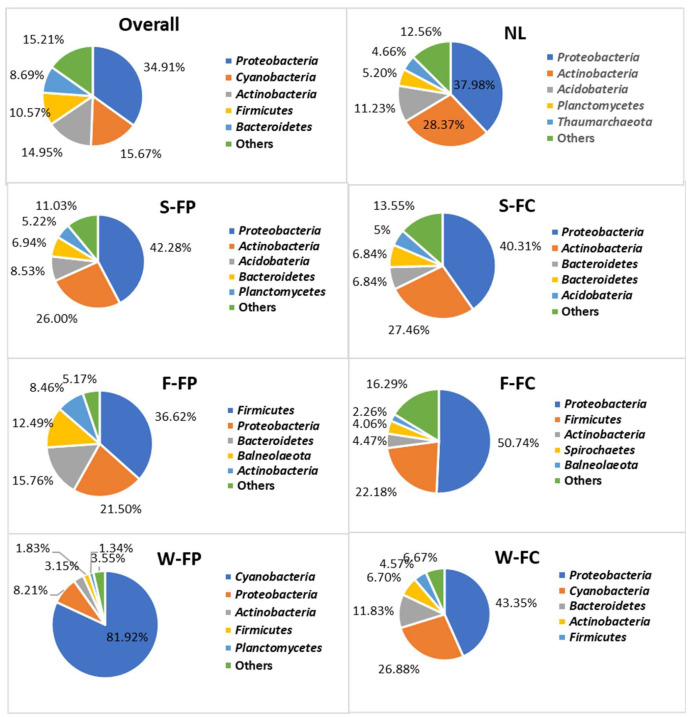
Relative abundance (%) of microbial communities at phylum level in natural land soil (NL), soil (S), feces (F) and water (W) from poultry (FP)/cattle (FC) farms.

Analysis of soil samples revealed that *Proteobacteria* and *Actinobacteria* were the dominant bacterial phyla, accounting for 37.98–42.28% and 26.07–28.37% of the total community, respectively (Figure 1). In animal manure samples, *Proteobacteria* and *Firmicutes* were the most abundant phyla. *Proteobacteria* constituted 50.74% of the microbial community in cattle manure, while *Firmicutes* accounted for 36.62% in poultry manure. Microbial profiling of farm water sources showed that bacterial community composition varied between cattle and poultry farms. *Proteobacteria* dominated cattle farm water (43.35%), whereas *Cyanobacteria* were overwhelmingly dominant in poultry farm water (81.92%). Other phyla detected across water samples included *Bacteroidetes*, *Actinobacteria*, and *Firmicutes*.

### 3.2. Antibiotic Resistant Genes (ARGs) in Cattle and Poultry Farms

**Figure 2:**
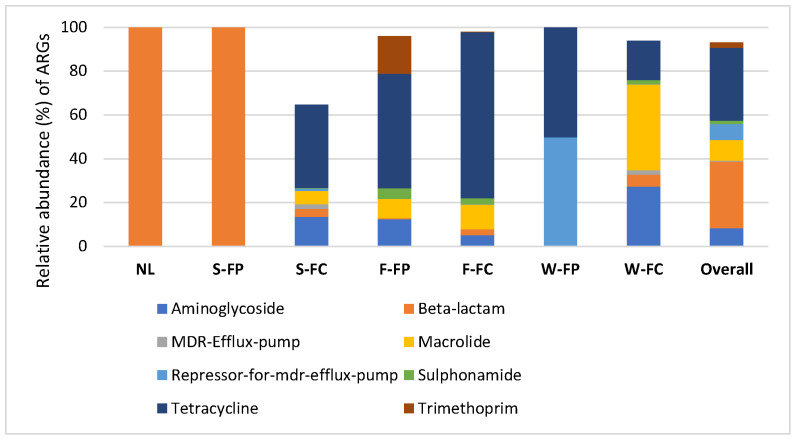
Relative abundance (%) of ARGs conferring resistance to predominant antibiotic classes in natural land soil (NL), soil (S), feces (F) and water (W) from poultry (FP)/cattle (FC) farms.

A total of 155 distinct antibiotic resistance genes (ARGs) conferring resistance to 31 classes of antimicrobials were identified across poultry and cattle farm samples. The most abundant ARG classes were those conferring resistance to tetracyclines (33.41%), beta-lactams (30.30%), macrolides (9.32%), aminoglycosides (8.39%), and repressor-for-multidrug-efflux-pump (7.29%) (Figure 2). Overall, tetracycline- and beta-lactam-associated ARGs were significantly more abundant than other resistance classes (*p* < 0.05) across sample types.

**Figure 3:**
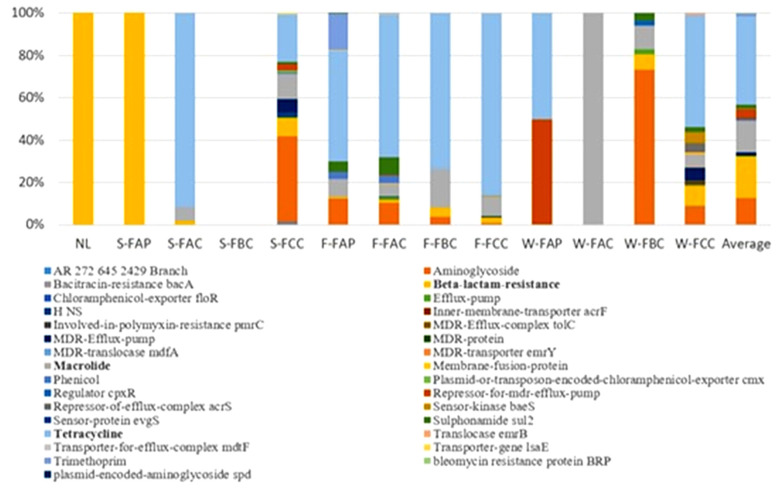
The relative abundance of top 31 antibiotic resistance genes (ARGs) in NL = natural land soil, S = soil, F = feces, W = water, FAP = poultry farm A, FAC = cattle farm A, FBC= cattle farm B, and FCC = cattle farm C.

The predominant tetracycline resistance genes included *tet40*, *tet44*, *tetO*, *tetQ*, *tetW*, and *tetX*, while *cfxA*, *pbp*, *blaOXA*, and *blaTEM* were the most common beta-lactam resistance genes. Aminoglycoside resistance was primarily associated with *aadA* and *aph*, macrolide resistance with *lnuC* and *ermF*, and MDR-efflux regulation with *crp* and *emrR*. In soil samples, tetracycline resistance genes were absent in poultry farm soil (0.00%) but were detected at 38.02% in cattle farm soil. Conversely, beta-lactam resistance genes were detected at 100% in natural land soil and poultry farm soil, compared to 3.5% in cattle farm soil. Aminoglycoside resistance genes were present in cattle farm soil (13.61%) but were not detected in natural land or poultry farm soils. Fecal samples from poultry and cattle farms contained high proportions of tetracycline resistance genes, accounting for 52.21% and 75.73%, respectively. Macrolide resistance genes were detected at 8.71% in poultry feces and 11.20% in cattle feces, while aminoglycoside resistance genes accounted for 12.48% and 5.20%, respectively. Water samples also showed distinct ARG profiles (Figure 3). In poultry farm water, tetracycline (50.19%) and MDR-efflux-pump-associated ARGs (49.81%) predominated. In cattle farm water, macrolide (39.19%), aminoglycoside (27.42%), and tetracycline (17.72%) resistance genes were most abundant.

### 3.3. Total Prevalence of Bacterial Community and Antimicrobial Resistance Genes (ARGs)

The average relative abundance of bacterial phyla was 8.83% in feces, 7.62% in soil, 4.91% in natural land soil, and 4.78% in water samples. The highest average relative abundance of ARGs and virulence genes (VGs) was detected in feces (19.92% and 18.85%, respectively), followed by water (2.40% and 3.61%), soil (1.80% and 2.58%), and natural land soil (0.16% and 0.44%) (Table 1). Overall, ARGs and VGs were consistently more abundant in animal farming environments than in natural land soil.

## 4. Discussion

The predominance of *Proteobacteria* and *Actinobacteria* in soil observed in this study aligns with previous reports identifying these phyla as major constituents of agricultural soils [25,26]. However, their relative abundances were slightly higher than those reported by [25], who documented *Proteobacteria* and *Actinobacteria* at 30–33% and 17–22%, respectively, in organic and conventional farming soils. Similar dominance patterns have also been reported in soils from urban community gardens and agricultural lands [10,32], suggesting that these phyla are consistently prevalent across diverse soil environments. Variations in phylum-level abundance among studies are likely driven by differences in land use, nutrient availability, and management practices. *Proteobacteria* are commonly associated with nutrient-rich and intensively managed soils, whereas Actinobacteria tend to thrive in nutrient-poor or organically managed systems [32,33]. Beyond their prevalence, the dominance of Proteobacteria and Actinobacteria suggests active involvement in key soil ecosystem functions, including carbon turnover, nitrogen cycling, and organic matter degradation, processes that may indirectly influence pathogen survival and the maintenance of antimicrobial resistance in agricultural soils.

In animal manures, the dominance of *Proteobacteria* and *Firmicutes* observed here corroborates earlier findings showing that manure microbial communities are strongly influenced by animal species, feeding practices, and manure handling [34,35]. The relative abundances detected in this study are comparable to reports of cattle and poultry manure compost containing approximately 41% and 38% of these phyla, respectively [36]. Nonetheless, other studies have shown that manure amendment and post-application environments can substantially alter microbial composition, with *Bacteroidetes* dominating in some poultry litter-amended soils [37]. This highlights the dynamic nature of manure-associated microbiomes and their sensitivity to environmental context. Such shifts following manure application may influence both the persistence of zoonotic pathogens and the redistribution of antimicrobial resistance genes in receiving soils. Microbial communities in farm water sources exhibited variation between poultry and cattle farm systems, reflecting differences in water management practices and environmental conditions. Prior studies have emphasized that agricultural water microbiota are shaped by environmental conditions and seasonal factors and play a critical role in preharvest food safety [38,39,40]. The marked dominance of *Cyanobacteria* in poultry farm water compared with *Proteobacteria* in cattle farm water likely reflects differences in nutrient loading, light availability, and water management practices. Given that farm water sources are often used for animal watering, cleaning, or irrigation, these microbial differences may have downstream implications for pathogen transmission and produce contamination.

Collectively, these findings indicate that farm management practices, animal source, storage conditions, and environmental factors jointly shape microbial community composition across soil, manure, and water. Of particular concern is the consistent dominance of *Proteobacteria* across all matrices, as this phylum includes clinically important pathogens such as *Escherichia coli*, *Klebsiella*, *Salmonella*, and *Pseudomonas.* These taxa are known reservoirs of antimicrobial resistance genes (ARGs) and virulence factors (VFs), and their abundance increases the potential for horizontal gene transfer and dissemination of resistance within farm environments and the food chain [25,41]. The high relative abundance of Proteobacteria may therefore serve as an ecological indicator of elevated resistome and virulome potential in livestock-associated environments.

Furthermore, the co-dominance of *Actinobacteria*, *Firmicutes*, and *Bacteroidetes* may contribute to the resilience and redundancy of the agroecosystem resistome. *Actinobacteria* are prolific antibiotic producers but also harbor resistance genes [42], while *Firmicutes* and *Bacteroidetes* are frequently associated with macrolide and aminoglycoside resistance [43]. This compositional diversity likely points out the broad distribution of ARGs observed and shows the capacity of agricultural systems to sustain antimicrobial resistance under varying environmental pressures [44]. All in all, these phyla form a functionally diverse microbial network capable of maintaining resistance traits even under fluctuating antibiotic exposure and environmental stressors.

### 4.1. Antibiotic Resistance Genes (ARGs) in Cattle and Poultry Farms

The dominance of tetracycline and beta-lactam resistance genes observed in this study reflects antibiotic usage patterns in animal production systems. Tetracyclines are the most extensively used antibiotics in livestock production, accounting for more than two-thirds of antimicrobials used in animals in the United States [15]. They are commonly administered to treat respiratory, gastrointestinal, and skin infections in both cattle and poultry [16,17]. The widespread detection of tetracycline resistance genes across feces, soil, and water samples suggests sustained selective pressure from tetracycline use in farming environments. Persistent exposure to sub-inhibitory concentrations of these compounds in the environment likely further promotes the maintenance and spread of tetracycline resistance determinants.

Differences in ARG distribution between cattle and poultry farm soils likely reflect variations in antibiotic dosing and animal physiology. Aminoglycosides, which are administered on a body-weight basis, were detected only in cattle farm soil. Higher body mass in cattle necessitates higher antibiotic doses, increasing environmental loading through excretion and promoting selective pressure for aminoglycoside resistance [45]. Similar explanations may account for other observed differences in ARG prevalence between poultry and cattle farms.

Despite regulatory restrictions prohibiting the use of tetracyclines as growth promoters in the United States (FDA, 2012), tetracycline resistance genes were highly abundant in cattle farm soil, feces, and water. This observation suggests continued therapeutic use and highlights the environmental persistence of tetracyclines. Previous studies have shown that animals excrete more than 75% of administered antibiotics unmetabolized, allowing them to accumulate in soil and water as biologically active compounds [18,46]. Tetracyclines are known to strongly adsorb to soil and manure and can persist for extended periods, thereby maintaining long-term selection for resistance [19,20]. These conditions create favorable environments for horizontal gene transfer mediated by mobile genetic elements such as plasmids, integrons, and transposons.

The detection of tetQ, tetW, tetO, and related genes is consistent with earlier studies reporting their prevalence in manure and wastewater from livestock farms [9]. Additionally, sul2, a sulfonamide resistance gene of clinical importance, was identified among the most abundant ARGs in fecal samples, in agreement with reports from livestock and poultry farm environments [41,47].

Macrolide resistance genes ranked third among detected ARG classes. Given that macrolides are critically important antibiotics in human medicine [48], their presence in animal production systems warrants particular concern. Genes such as ermB and ermF have been widely reported in livestock-associated environments, including manure-amended soils, wastewater, and fecal samples, underscoring their environmental dissemination potential [11,41,49,50].

Beta-lactam resistance genes were the second most prevalent ARG class identified. While previous studies reported higher beta-lactam resistance gene abundance in poultry manure compared to cattle manure [26], the present study observed higher relative abundance in cattle feces. The near-ubiquitous presence of beta-lactam resistance genes in natural land and poultry farm soils is consistent with prior findings from urban and agricultural soils [10]. This widespread distribution suggests that beta-lactam resistance genes are well established in environmental reservoirs beyond direct antibiotic application sites.

Aminoglycoside resistance genes, particularly *aadA* and *aph*, were widely distributed across sample types. These genes have been commonly detected in manure, compost, irrigated soils, and wastewater [51,52]. Given the importance of aminoglycosides in treating severe human infections, including multidrug-resistant tuberculosis and Gram-negative bacterial infections, their environmental persistence poses significant public health risk [53,54].

Finally, the detection of MDR-efflux-pump-associated genes across cattle farm soil, feces, and water is particularly concerning. MDR-efflux systems are key contributors to multidrug resistance in clinical pathogens and have been shown to play a central role in resistome development under antibiotic pressure [55,56]. Their presence in agricultural environments highlights the potential for emergence and dissemination of multidrug-resistant bacteria with implications for both animal and human health. Efflux-mediated resistance further complicates mitigation efforts, as it can confer cross-resistance to multiple antibiotic classes and disinfectants.

### 4.2. Total Prevalence of Bacterial Communities and Antimicrobial Resistance Genes

The elevated abundance of ARGs and VGs in animal farming environments compared with natural land soil reflects the strong influence of human and animal activities. Studies conducted in minimally human-impacted environments, such as pristine Antarctic soils, have demonstrated that resistance genes in such systems primarily confer resistance to naturally occurring antibiotics rather than synthetic compounds [27]. In contrast, agricultural soils commonly harbor resistance genes to both natural and synthetic antibiotics, indicating anthropogenic influence [57]. This contrast underscores the role of agricultural intensification in reshaping environmental resistomes.

Farm animals play a central role in shaping the microbial and resistome dynamics of agricultural ecosystems. Routine exposure to antibiotics for disease treatment results in substantial excretion of unmetabolized compounds via urine and feces, which subsequently enter soil and water matrices [18]. These residues exert selective pressure on surrounding microbial communities, promoting the proliferation and persistence of ARGs and VGs [58]. In addition, high microbial densities in farm environments increase opportunities for resistance gene exchange among commensal, environmental, and pathogenic bacteria.

Therefore, the higher abundance of ARGs and VGs observed in feces, soil, and water from animal farms relative to natural land soil is a consequence of the complex and intensively managed nature of farming systems. These findings highlight the role of livestock production environments as important reservoirs and dissemination points for antimicrobial resistance, with potential implications for environmental health, food safety, and public health. These results emphasize the need for integrated management and surveillance strategies that address antimicrobial use, waste handling, and environmental pathways within a One Health framework.

## 5. Conclusions

In conclusion, this study depicted that farming production systems could be an important environmental reservoir of antimicrobial resistance. Identification of clinically significant ARGs in the farming environment suggests a strong potential for horizontal transfer of ARGs between different farm components, including soil, feces, and water. The data from our study can be used to understand the ARGs and VGs dissemination between and among microbial communities in cattle and poultry farming production. The data from this study is proof that AMR poses a threat not only in clinical setups but also in agroecosystems and food safety. It is a great challenge for the food animal producers to increase animal production to meet the global protein demand and simultaneously minimize ARG and VG threats at different food chain levels. Therefore, the data obtained from this study would be beneficiary to develop an antimicrobial resistance mitigation strategy in the farming production system. Examples of these strategies include improved manure management, targeted reduction in antibiotic use, treatment of farm water sources, and implementation of best management practices to limit environmental dissemination of resistance genes. This can be helpful to limit drug-resistant infections in both humans and food animals. While the limited number of farms and single-state focus restrict national generalizability, the findings provide important baseline data to inform future multi-state and longitudinal investigations. Further studies are necessary to assert microbial taxonomic distribution and horizontal gene transfer between microbial communities from different farm components, including soil, feces, and water, within poultry and cattle production systems.

## Figures and Tables

**Table 1:** Relative abundance (%) of phylum, antibiotic resistance (AR) and virulence (VR) genes functionally in natural land soil (NL), soil (S), feces (F) and water (W) from farm of poultry (FP)/cattle (FC).

Category	NL	S-FP	S-FC	Soil Average	F-FP	F-FC	Feces Average	W-FP	W-FC	Water Average
Phylum (*n* = 22)	**4.91** ^bx^	6.43	8.80	7.62 ^abx^	9.59	8.08	8.83 ^ay^	2.10	7.45	4.78 ^bx^
ARGs (*n* = 155)	**0.16** ^cy^	0.16	3.44	1.80 ^by^	21.77	18.06	19.92 ^ax^	0.49	4.31	2.40 ^by^
VGs (*n* = 147)	**0.44** ^cy^	1.62	3.54	2.58 ^by^	23.53	14.17	18.85 ^ax^	0.15	7.06	3.61 ^by^

Values represent mean relative abundance. Different superscript letters (a–c) indicate statistically significant differences among sample types within the same column, while different superscript letters (x–y) indicate statistically significant differences among categories within the same row (*p* < 0.05; one-way ANOVA).

## Data Availability

The original contributions presented in this study are included in the article. Further inquiries can be directed to the corresponding author.

## Abbreviations

The following abbreviations are used in this manuscript:

VFs	Virulence factors
ARGs	Antibiotic Resistance Genes
AMR	Antimicrobial resistance
VRs	Virulence genes
ARB	Antibiotic-resistant bacteria
BMP	Best management practice
IACUC	Institutional Animal Care and Use Committee
EMB	Eosin methylene blue (agar)
DNA	Deoxyribonucleic acid
